# Case report: contradictory genetics and imaging in focal congenital hyperinsulinism reinforces the need for pancreatic biopsy

**DOI:** 10.1186/s13633-020-00086-2

**Published:** 2020-08-31

**Authors:** Daphne Yau, Ria Marwaha, Klaus Mohnike, Rakesh Sajjan, Susann Empting, Ross J. Craigie, Mark J. Dunne, Maria Salomon-Estebanez, Indraneel Banerjee

**Affiliations:** 1 Department of Paediatric Endocrinology, Royal Manchester Children’s Hospital, Manchester, M13 9WL UK; 2 Department of Pediatrics, University of Saskatchewan, Royal University Hospital, 103 Hospital Drive, Saskatoon, Saskatchewan S7N 0W8 Canada; 3 Department of Paediatrics, Otto von Guericke University Magdeburg, 39106 Magdeburg, Germany; 4 Nuclear Medicine Centre, New Saint Mary’s Hospital, Manchester University Foundation Trust, Manchester, M13 9WL UK; 5 Department of Paediatric Surgery, Royal Manchester Children’s Hospital, Manchester, M13 9WL UK; 6 Faculty of Biology, Medicine and Health, University of Manchester, Manchester, M13 9PL UK

**Keywords:** Focal congenital hyperinsulinism, Fluorodopa F 18, Positron emission tomography computed tomography, KATP channels, Kir6.2 channel

## Abstract

**Background:**

Congenital Hyperinsulinism (CHI) is an important cause of severe hypoglycaemia in infancy due to excessive, dysregulated insulin secretion. In focal CHI, a localised lesion within the pancreas hypersecretes insulin and, importantly, hypoglycaemia resolution is possible through limited surgical resection of the lesion. Diagnosis of focal CHI is based on a crucial combination of compatible genetics and specialised imaging. Specifically, a focal lesion arises due to a paternal mutation in one of the ATP-sensitive potassium channel genes, *KCNJ11* or *ABCC8*, in combination with post-zygotic loss of maternal heterozygosity within the affected pancreatic tissue. 6-[18F]Fluoro-L-3,4-dihydroxyphenylalanine (^18^F-DOPA) positron emission tomography (PET)/computed tomography (CT) imaging is used to detect and localise the lesion prior to surgery. However, its accuracy is imperfect and needs recognition in individual case management.

**Case presentation:**

We report the case of an infant with hypoglycaemia due to CHI and a paternally inherited *KCNJ11* mutation, c.286G > A (p.Ala96Thr), leading to a high probability of focal CHI. However,^18^F-DOPA PET/CT scanning demonstrated diffuse uptake and failed to conclusively identify a focal lesion. Due to unresponsiveness to medical therapy and ongoing significant hypoglycaemia, surgery was undertaken and a small 4.9 × 1.7 mm focal lesion was discovered at the pancreatic neck. This is the second case where this particular *KCNJ11* mutation has been incorrectly associated with diffuse ^18^F-DOPA uptake, in contrast to the correct diagnosis of focal CHI confirmed by pancreatic biopsy.

**Conclusions:**

Identifying discrepancies between genetic and imaging investigations is crucial as this may negatively impact upon the diagnosis and surgical treatment of focal CHI. This case highlights the need for pancreatic biopsy when a strong suspicion of focal CHI is present even if ^18^F-DOPA imaging fails to demonstrate a discrete lesion.

## Background

Congenital Hyperinsulinism (CHI) is an important cause of severe hypoglycaemia in infancy due to excessive, dysregulated insulin secretion [1]. In CHI, the most common genetic aetiology is a mutation in one of the potassium ATP (K_ATP_) channel genes, *ABCC8* or *KCNJ11*. This channel links glucose metabolism to insulin release in the pancreatic beta-cell. Recessive K_ATP_ mutations in the homozygous or compound heterozygous state cause diffuse CHI, in which beta-cells throughout the entire pancreas are abnormal and hypersecrete insulin [2]. Diffuse CHI due to K_ATP_ mutations is typically unresponsive to medication, requiring subtotal pancreatectomy to achieve normoglycaemia.

In focal CHI, another form of CHI that is also generally medication-unresponsive, only a small region of the pancreas is affected by pathology [1]. The focal lesion occurs due to a paternally inherited mutation in *ABCC8* or *KCNJ11* combined with post-zygotic loss of maternal heterozygosity within the affected tissue. The latter causes clonal expansion of endocrine rich tissue due to the loss of cell cycle repressor genes that are expressed from the maternal allele [2]. Excess insulin results from the clonally expanded, abnormal beta-cells expressing the mutated ATP-sensitive potassium channel. Focal CHI can be cured by limited resection, compared with more extensive pancreatectomy, which is associated with significant risk of lifelong diabetes and exocrine insufficiency.

Distinguishing focal CHI from other forms is therefore critical, and its diagnosis is based on a crucial combination of compatible genetics (i.e. paternal mutation in *KCNJ11* or *ABCC8*) and 6-(18F)Fluoro-L-3,4-dihydroxyphenylalanine (^18^F-DOPA) positron emission tomography (PET)/CT imaging which localises the lesion [1, 2]. Uptake of ^18^F-DOPA by pancreatic beta-cells occurs due to expression of aromatic amine decarboxylase (AADC), resulting in conversion of L-DOPA to dopamine and sequestration of the radiotracer within the beta-cells [3, 4]. Focal lesions are distinguishable from the remainder of the pancreas due to uptake and sustained retention of the radiotracer by the hyperplastic, clonally-expanded beta-cells within the lesion, in contrast to the rest of the pancreas [4]. The mechanism for this remains unclear.

^18^F-DOPA PET/CT represents a significant advance over the invasive and technically challenging technique of pancreatic arterial calcium stimulation with hepatic venous sampling previously used to identify focal lesions [4]. However, while this modality remains the investigation of choice to non-invasively identify a focal CHI lesion prior to surgery, with greater experience, it has been found to be less sensitive in detecting focal lesions than originally reported [5, 6]. We report a case in which a paternally inherited *KCNJ11* mutation was associated with diffuse ^18^F-DOPA uptake but later confirmed to have focal CHI at the time of surgery.

## Case presentation

A male infant was born at 39 weeks’ gestation with a birth weight of 4.34 kg (98–99.6th percentile) after an uncomplicated pregnancy and normal delivery. Profound hypoglycaemia was noted with poor feeding and irritability in the early neonatal period. He required an elevated glucose infusion rate of 15.4 mg/kg/min to achieve normoglycaemia. Consistent with this, CHI was confirmed with raised insulin and c-peptide at the time of hypoglycaemia (glucose 1.8 mmol/L, insulin 140 pmol/L, c-peptide 741 pmol/L). There were no dysmorphic features or other medical problems to suggest a syndromic aetiology. He was unresponsive to medical therapy with persistent need for high dextrose infusion despite maximum doses of diazoxide and octreotide, the mainstays of medical treatment, which act by inhibiting insulin release.

**Fig. 1:**
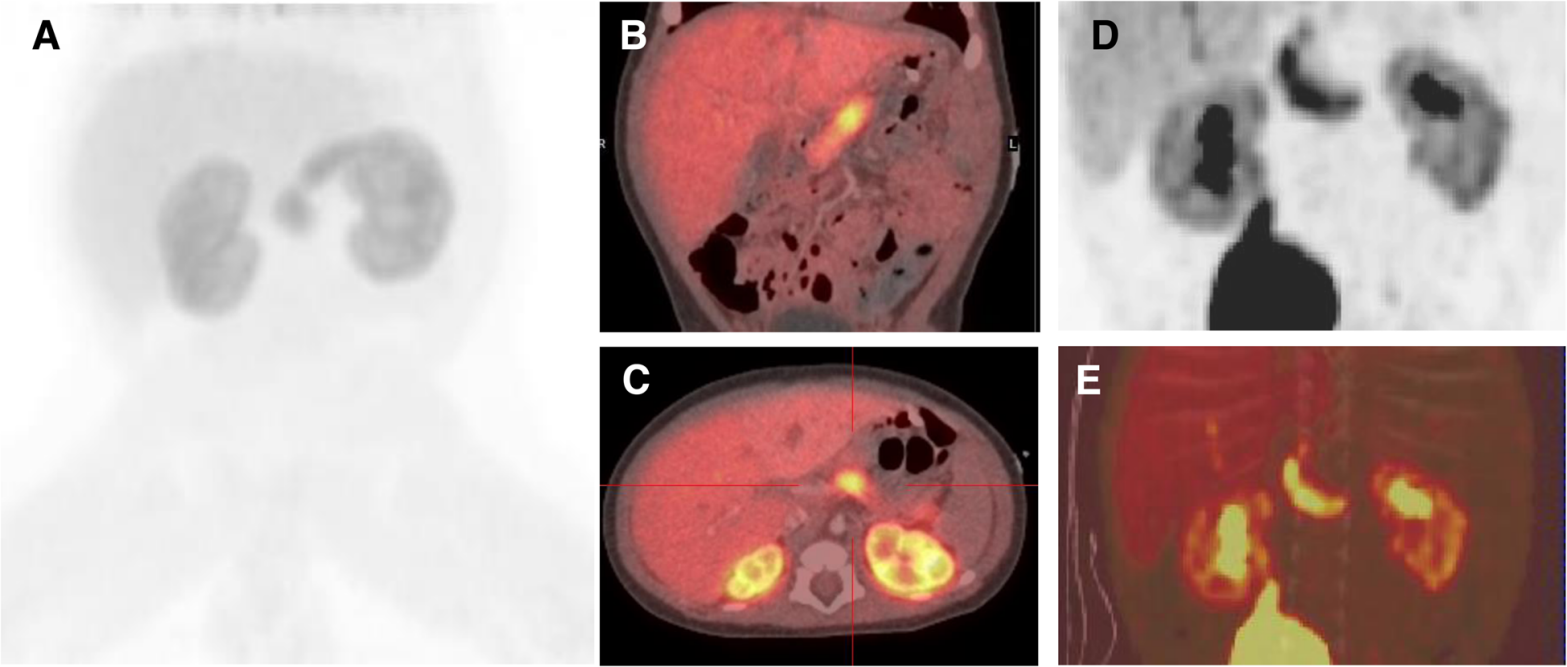
^18^F-DOPA PET/CT Pancreatic Imaging. ^18^F-DOPA PET/CT imaging in the case described demonstrates diffuse pancreatic uptake (**a**-**c**). A coronal maximal intensity projection (MIP) image with OSEM reconstruction is shown at 30–45 min after radionucleotide injection (**a**) with merged coronal (**b**) and axial (**c**) PET and CT images. The previous patient with the same *KCNJ11* mutation [7] demonstrated similar findings on ^18^F-DOPA PET-CT scanning with diffuse uptake throughout the pancreas at 44 min post-injection on both MIP and merged PET-CT coronal images (**d**, **e**)

Genetic testing revealed a paternally inherited mutation in *KCNJ11*, c.286G > A (p.Ala96Thr), suggesting the presence of focal CHI. This mutation has previously been described as recessive and a cause for focal CHI in another patient [7]. However, ^18^F-DOPA PET/CT scanning demonstrated diffuse uptake throughout the entire pancreas (Fig. 1A-C). Both ordered subset expectation maximization (OSEM) and ultra-high definition (UHD) reconstruction were performed on the PET images and standardised uptake values (SUV) measurements were obtained at each time point (5–15 min, 15–30 min, 30–45 min, 45–60 min and 60–80 min) at the following sites: pancreatic head, body, tail, and region of most avid uptake (Table 1). In addition, maximum intensity projection (MIP) images were visually inspected for localised increased radiotracer concentrations, but there was no obvious localisation of a focal lesion.

**Table 1:** SUV from both OSEM and UHD reconstruction of the ^18^F-DOPA PET Imaging shown up to a post injection duration of 60 min

	Reconstruction Format	Time Post^ 18^F-DOPA Administration (mins)			
		5–15	15–30	30–45	45–60
Region of most avid uptake	OESM	6.0	6.8	6.7	6.9
	UHD	9.2	10.9	10.4	10.7
Head	OESM	6.7	6.5	6.5	6.5
	UHD	9.2	9.8	9.3	9.2
Body	OESM	6.7	6.6	6.6	6.0
	UHD	9.8	9.4	8.7	8.5
Tail	OESM	5.7	5.8	6.1	5.9
	UHD	8.4	9.0	9.6	8.5
Mean	OESM	6.3	6.3	6.4	6.1
	UHD	9.1	9.4	9.2	8.7

The uptake was quantified by calculating SUV ratios for the area of most avid intake, which was in the proximal body, at 45–60 min (Table 1). The maximum SUV value was normalized to the mean of the entire pancreas for both OESM and UHD reconstruction, giving ratios of 6.9/6.1 = 1.1 and 10.7/8.7 = 1.2 respectively, whereas a ratio of > 1.5 is generally considered predictive of focal disease.

The authors of the previous patient with the same *KCNJ11* mutation and focal CHI were contacted to share their experience to guide clinical management [7]. Similar scan findings to our case were noted (Fig. 1D-E). As this previous child’s condition had been unstable with no response to medical therapy, surgery had been performed and a focal lesion was identified. Hypoglycaemia resolved after surgery.

**Fig. 2:**
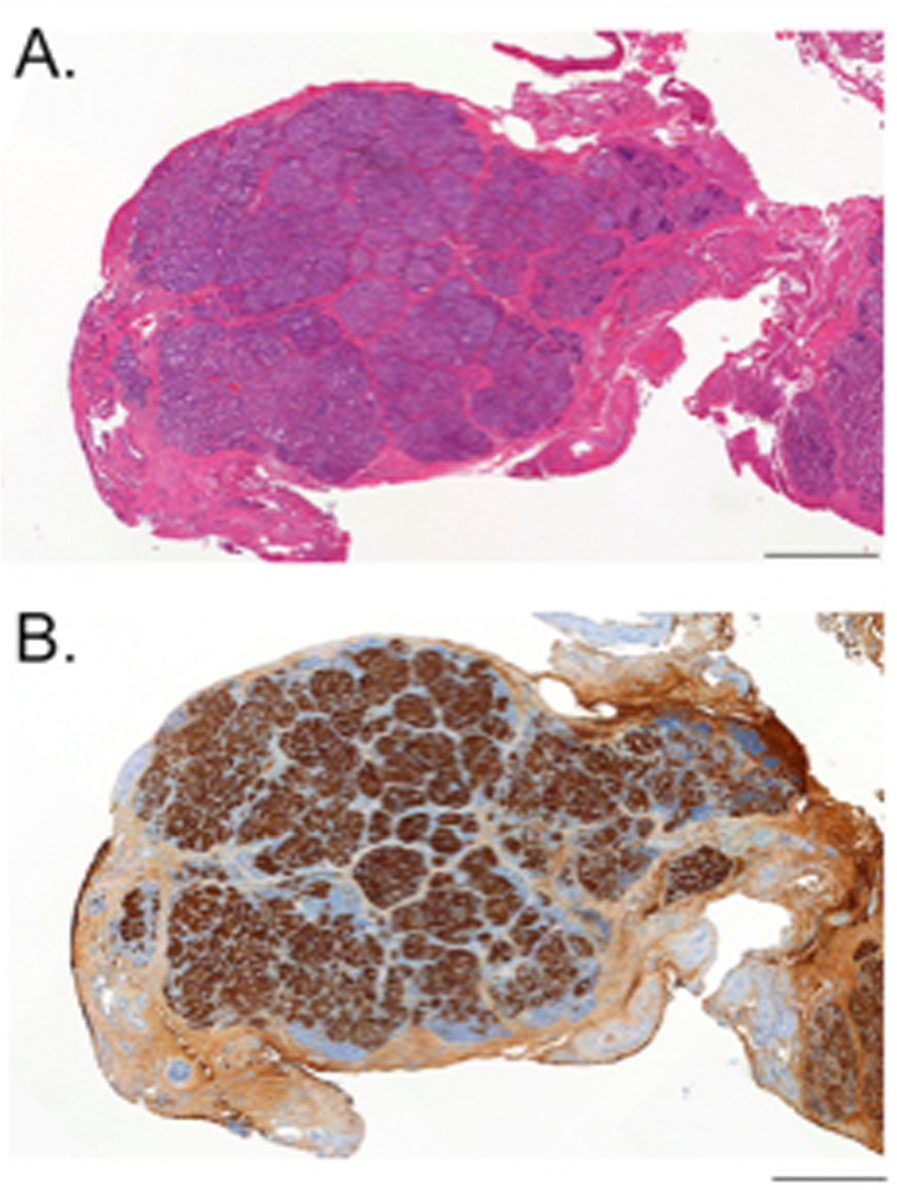
Focal CHI Lesion Histology. (**a**) Haematoxylin & eosin and (**b**) insulin staining of the resected pancreas revealed a 4.9 × 1.7 mm nodular lesion consistent with the presence of a focal lesion. Size marker is equivalent to 0.5 mm

Given this outcome, the decision was made to proceed to pancreatic biopsy, leading to the identification of a 4.9 mm by 1.7 mm lesion at the pancreatic neck. The histology demonstrated islands of endocrine cells, predominantly insulin and proinsulin positive, interspersed by bands of fibrous tissue and some acinar cells, consistent with a focal lesion (Fig. 2). Additional biopsy of the pancreatic tail showed normal pancreatic tissue without evidence of hyperplasia. After resection of the focal lesion, hypoglycaemia resolved and the patient was able to maintain an age-appropriate duration of fasting on regular feeds.

## Discussion and conclusions

We describe the case of patient with focal CHI and discordant genetic and imaging findings. Although the patient possessed a paternally inherited K_ATP_ mutation, diffuse uptake was seen on ^18^F-DOPA PET/CT scan and ultimately, pancreatic biopsy was required to identify and confirm the focal lesion.

While the inheritance of a recessive paternal K_ATP_ mutation is a key feature in the diagnosis of focal CHI, the presence of pancreatic loss of heterozygosity and histologic confirmation of a focal lesion cannot be determined without biopsy of the tissue. While ^18^F-DOPA PET/CT is used to identify and localise focal CHI lesions prior to surgery [1, 2], it is important to recognise the possibility of discordant outcomes, as demonstrated by this case.

The reason for under-detection of focal lesions is unclear. Given the postulated mechanism of AADC beta-cell expression, and sequestration of L-DOPA following conversion to dopamine, localised tracer uptake by the clonally-expanded beta-cells would be expected to occur for all focal lesions. One potential mechanism may be related to lesion size [6]. False negative results have been attributed to both large and small focal lesions, with the smallest reported lesion detected by ^18^F-DOPA PET/CT being 5 × 4 mm [6, 8]. It is possible that the lesion in our patient was too small at 4.9.× 1.7 mm for detection. Of note, this lesion is one of the smallest lesions identified at our centre.

Similar discordance in imaging findings have been observed in a separate case with the same mutation [7]. This raises the possibility of a mutation specific imaging outcome, although without a mechanistic explanation. Alternatively, the findings could be purely coincidental. Regardless, this case highlights the need for intra-operative pancreatic biopsy if focal CHI is strongly suspected but ^18^F-DOPA PET/CT scanning fails to identify a well delineated focal lesion. Our case reinforces the importance of pancreatic biopsy in order to avoid unnecessary extensive pancreatectomy in focal CHI and the attendant future risks of diabetes and exocrine insufficiency.

## Data Availability

Data sharing is not applicable to this article as no datasets were generated or analysed during the current study.

## Abbreviations

AADC: aromatic amine decarboxylase
CHI: congenital hyperinsulinism
CT: computed tomography
^18^F-DOPA: 6-(18F)Fluoro-L-3,4-dihydroxyphenylalanine
PET: positron emission tomography
